# Cardiac Disease Patterns in Northern Malawi: Epidemiologic Transition Perspective

**DOI:** 10.2188/jea.JE2008006

**Published:** 2008-10-01

**Authors:** Elsayed Z. Soliman, Hadge Juma

**Affiliations:** 1 Epidemiological Cardiology Research Center (EPICARE), Department of Epidemiology and Prevention, Wake Forest University School of Medicine, Winston-Salem, North Carolina, USA; 2 Departments of Medicine, Mzuzu Central Hospital, Mzuzu, Malawi

**Keywords:** Malawi, Cardiovascular, Epidemiologic Transition

## Abstract

**Background:**

Cardiovascular disease (CVD) is a strongly emerging problem in developing countries. The documentation and prediction of CVD patterns are important for policy makers if actions are to be taken to curb this problem. We aimed to document the current CVD patterns in Malawi, and associate these patterns to the theory of epidemiologic transition as a means of predicting future CVD patterns.

**Methods:**

We retrospectively analyzed the data recorded in the register of the cardiac clinic in Mzuzu Central Hospital-the only cardiac clinic run by a cardiologist in Malawi-from 2001 through 2005. The findings were interpreted in the context of the epidemiologic transition theory.

**Results:**

Out of the 3908 new Malawian patients included in the 5-y period register, 34% had valvular heart disease (mainly rheumatic heart disease (RHD)); 24%, hypertensive heart disease; 19%, cardiomyopathies; and 14%, pericardial diseases. The other CVD patterns included congenital heart disease and arrhythmias, each representing 4% of the registered patients. Among the 1% comprising other CVD patterns, 3 cases were documented to have coronary heart disease, all of which happened in 2005.

**Conclusion:**

Malawi is in the stage of receding pandemics, which is characterized by CVD patterns predominated by RHD, cardiomyopathies, and hypertensive heart disease. However, continuous observation is required to detect signs of emerging “degenerative-related” CVD patterns, which is another stage in the epidemiologic transition.

## INTRODUCTION

Cardiovascular disease (CVD) is the biggest cause of death globally and is projected to remain the leading cause of death. An estimated 17.5 million people died from CVD in 2005, which constitutes 30% of all global deaths. Around 80% of these deaths occurred in low- and middle-income countries.^1^^,^^2^

Urbanization, globalization, and ageing of populations are the main causes of the cardiovascular epidemic.^3^ All these 3 factors are in a state of constant change, which is reflected in the subsequent continuous change in the CVD patterns. Hence, monitoring the epidemiologic transition by first documenting the current disease patterns is important for future comparisons, if action is to be taken to curb this cardiovascular epidemic.

Similar to most Sub-Saharan countries, there is a lack of proper statistical data on the patterns of CVD in Malawi, which is a densely populated country located in southeastern Africa. This is mainly due to the lack of the necessary expertise required to classify disease patterns and the lack of resources required to conduct large-scale formal national surveys. Although the epidemiology of communicable diseases such as HIV/AIDS, which now affects 10-15% of the population in Malawi,^5^ have been extensively studied, data on the epidemiology of non-communicable diseases, especially CVD, is not enough. Hence, for the first time, this study documents the CVD patterns in the Northern Region of Malawi, which constitutes more than one-third of the total land area of the country.

## METHODS

This is a retrospective analysis of the data from the register of the outpatient cardiology clinic of Mzuzu Central Hospital during the period from January 2001 through August 2005. Mzuzu Central Hospital is the only tertiary health facility in the Northern Region of Malawi, which has a population of over 1.5 million, out of the 12 million overall population of the country.^5^ The cardiac clinic at Mzuzu Central Hospital was the only cardiac clinic run by a cardiologist in Malawi from January 2001 through August 2005. All patients visiting the clinic were registered by the cardiologist or one of his medical assistants in a register that was always kept at the clinic.

The data collected included basic demographic data (age, sex, and ethnicity if not African), the observations made during physical examinations, and the results of electrocardiography (ECG), X-ray, and echocardiography, if available. A summary of the important laboratory findings was also included in the register. The International Classification of Diseases and Related Health Problems (ICD-9) was used for classification of the cases. We used the final diagnosis of each case in this analysis.

Hypertension was diagnosed in the case of an elevation of systolic blood pressure greater than 140mmHg, diastolic blood pressure greater than 90mmHg, or use of antihypertensive therapy. Hypertension was classified as being complicated if there were signs of end organ damage (chronic kidney disease, history of stroke, echo-diagnosed left ventricular hypertrophy, history of pulmonary edema, or heart failure).

Dilated cardiomyopathy was diagnosed if echocardiography revealed dilatation of both chambers of the heart (mainly the left ventricle) with ejection fraction below 50% and symptoms of systemic and/or pulmonary congestion.

Pericardial effusion caused by tuberculosis (TB) was diagnosed if the patient had a documented history of TB with the presence of pericardial effusion (diagnosed by echocardiography) in the absence of any other causes of pericardial effusion.

CVD categories were tabulated according to pattern, and infrequent patterns, defined as patterns reported 2 times or less in the 5-year period of registering, were placed under “others” in each category. In order to ensure homogeneity of the results, non-Malawians or Malawian citizens of non-African origin were excluded from the analysis. Patients suffering from conditions with a non-cardiac etiology or follow-up patients (repeated patients) were also excluded from the analysis. Patients who were registered were referred from either other clinics and hospitals or the in-patient department for evaluation before discharge, or they were self-referred.

The data collected on CVD patterns were interpreted within the context of the epidemiological transition theory described by Omran.^6^ According to Omran, the epidemiologic transition of diseases can be summarized into 4 stages: stage of pestilence and famine (characterized by rheumatic heart disease (RHD), infectious diseases, nutritional diseases, and cardiomyopathies), stage of receding pandemics (which includes hypertensive heart disease), stage of degenerative and man-made diseases (which includes coronary heart disease (CHD) at a relatively young age), and finally, the stage of delayed degenerative diseases (which includes stroke and CHD at older ages).

The study protocol has been approved by the Institutional Review Board of Mzuzu Central Hospital.

## RESULTS

In the period from January 2001 through August 2005, 3908 new Malawian patients with a diagnosis related to cardiac diseases were registered in the clinic, among whom, 6.3% were referred from the in-patient department (i.e., they had been recently admitted to in-patient wards). The age of the study population ranged from 2 months to 82 years with a mean and standard deviation of 39.9 ± 32.4 years, and a majority were females (58.9%).

**Figure 1.**
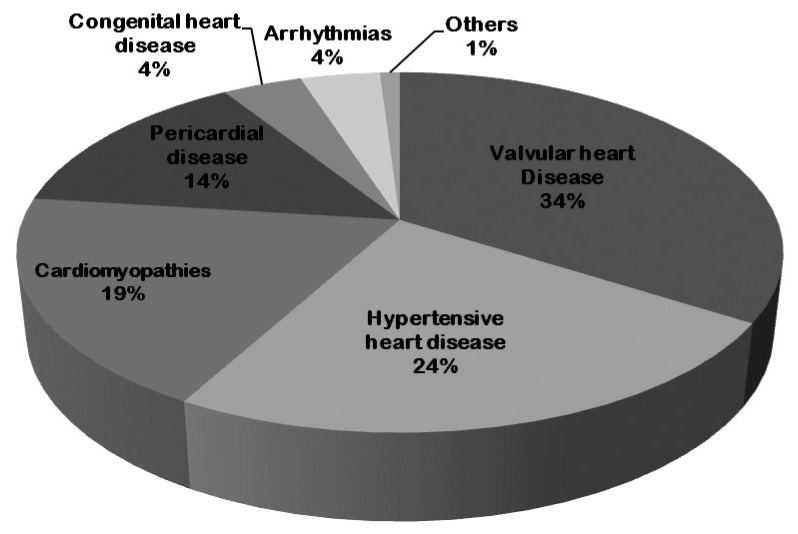
Proportional distribution of CVD patterns in the Northern Region of Malawi in the period from 2001 through 2005

Table 1 and Figure 1 show the frequency and the proportionate distribution of different CVD patterns respectively. The most common cardiac lesions were valvular heart disease (34%), which was followed by hypertensive heart disease (24%), cardiomyopathy (19%), and pericardial diseases (14%). RHD, dilated cardiomyopathy, and TB were the major causes of valvular heart disease, cardiomyopathies, and pericardial diseases respectively. During the 5-year registering period, there was no notable decrease in the trend of any of the CVD patterns reported in this study.

**Table 1.** Cardiovascular disease patterns in the Northern Region of Malawi in the period from 2001 through 2005

CVD pattern and demographic data	n = 3908
Age in years (mean ± SD)	39.9 ± 32.4
Females (%)	2299 (58.9%)
Referred from the in-patient department (%)	246 (6.3%)

Valvular heart disease	1315
Rheumatic Heart Disease	1176
Predominantly MR^*^	363
Predominantly MS^†^	204
Predominantly AR^‡^	161
Predominantly AS^§^	11
Multivalvular	437
Mitral valve prolapse	139

Hypertensive heart disease	945
Complicated hypertension	548
End-stage renal failure	78
Stroke	96
Echo-diagnosed LVH^||^	235
Hypertensive heart failure and/or pulmonary edema	107
Others	32
Uncomplicated hypertension	397

Cardiomyopathies	726
Dilated cardiomyopathy	720
Restrictive cardiomyopathy	3
Hypertrophic cardiomyopathy	3

Pericardial disease	553
Pericardial effusion	503
Tuberculosis effusion	492
Malignant	5
Uremic	3
Cardiac tamponade	3
Constrictive pericarditis	47

Congenital heart disease	173
Ventricular septal defect	78
Atrial septal defect	7
Patent ductus arteriosus	19
Pulmonary stenosis	11
Tetralogy of Fallot	13
Others	45

Arrhythmia	142
Atrial ectopics	57
Ventricular ectopics	23
Supraventricular tachycardia	4
Atrial fibrillation (lone)	27
Symptomatic bradycardia (possible SSSS)	7
Advanced degree atrioventricular block	11
Others	13

Other CVD patterns	54
Endocarditis	4
Deep venous thrombosis	7
Pulmonary embolism	3
Myocardial infarction	3
Others	37

^*^: mitral regurgitation; ^†^: mitral stenosis; ^‡^: aortic regurgitation; ^§^: aortic stenosis; ^||^: left ventricular hypertrophy

Patients with echocardiographic evidence of both mitral valve prolapse (MVP) and RHD were listed under RHD. Although MVP is benign and sometimes considered a normal variant, diagnosing MVP was important for differentiation of MVP patients from patients with other causes of murmur, especially RHD, who require regular penicillin injections for prophylaxis against recurrent rheumatic valvular lesions.

Complicated hypertension was more common than uncomplicated hypertension (58% vs. 42%). The increased symptoms in patients with complicated hypertension might be the major reason why they seek medical advice, which could explain their increased number compared to patients with uncomplicated hypertension.

Congenital heart disease was diagnosed in 4% of the patients. It was difficult to exactly describe the congenital abnormality in 26% of the patients with congenital heart disease since the diagnosis was based only on noninvasive diagnostic tools that included physical examination, X-Ray, and echo-Doppler.

Four percent of the patients presented with signs and symptoms related to arrhythmia. Although arrhythmia was a common finding in most patients, this 4% refers to the percentage of patients in whom arrhythmia was the main reason for visiting the cardiac clinic.

During the 5-year period of registering, only 3 patients had ECG-diagnosed CHD based on the presence of old anteroseptal myocardial infarction (QS in leads V1 through V4) in 2 patients and an old inferior myocardial infarction in the other (pathological Q wave in lead II with QS in leads III and aVF). These 3 patients were all diagnosed in 2005; prior to this, we were unable to find a single case of documented myocardial infarction.

From the reported CVD patterns, it seems like Malawi is in the stage of receding pandemics, which is characterized by CVD patterns predominated by RHD, cardiomyopathies, and hypertensive heart disease. However, there are signs of emerging degenerative-related CVD patterns-another stage in the epidemiologic transition.

## DISCUSSION

The theory of epidemiologic transition focuses on the complex change in the patterns of health and disease and on the interactions between these patterns and their demographic, economic, and sociologic determinants and consequences. An epidemiologic transition has occurred in parallel to the demographic and technologic transitions in the now developed countries of the world and is still underway in less developed countries. The epidemiologic transition, wherein control of non-communicable diseases allows most of the population to reach the age where CVD manifests itself, provides the basis for the prediction models of a CVD epidemic.^6^

In Malawi, even though communicable diseases are still not completely under control, life expectancy has been improving over the last few years. The life expectancy is currently 45 years and is expected to increase to 53 years in 2020.^7^ This is by all means a significant increase in the Malawian life expectancy, which was under 40 years prior to 2000.^5^ This increase in life expectancy will definitely have an impact on the dynamics of the epidemiologic transition in Malawi, which will reach a stage where more CVD patterns are expected to appear. For the purpose of monitoring and possibly intervening in the epidemiologic transition in Malawi, it is first important to document the current CVD patterns, which is the main purpose of this study.

The important features of this study are as follows: First, it is the first documentation of CVD patterns in Malawi conducted by a cardiologist. Second, the cardiac clinic at Mzuzu Central Hospital is the only clinic in the country equipped with a color echo-Doppler machine that is backed up by strong radiology and laboratory departments. Third, although this study is mainly concerned with patterns rather than the prevalence of CVD, considering that Mzuzu Central Hospital is the only tertiary care facility in the Northern Region of Malawi, it is expected that a significant proportion of the actual number of cardiac diseases in the Northern Region have been registered in the clinic. Fourth, this study being a retrospective analysis of the data already recorded in the cardiac clinic register, it can be easily repeated in the future for monitoring the trends of CVD patterns.

Furthermore, the period from 2001 through 2005, i.e., the survey period, is especially important because it witnessed the widespread use of highly active antiretroviral therapy (HAART) for HIV/AIDS. The introduction of HAART has significantly modified the course of HIV/AIDS. Longer survival of HIV patients is expected to be accompanied by an increase in the probability of developing CVD.^8^ Additionally, HAART regimens, especially those involving protease inhibitors, have been shown to cause metabolic changes that are associated with an increased risk of CVD (CHD and stroke) in the general population, creating an intriguing clinical scenery.^9^^,^^10^ Although it would be interesting to compare the current CVD patterns with those present prior to widespread HIV/AIDS infections, unavailability of such data remains an obstacle.

The key result of this study is that, currently, the CVD patterns in Malawi are mainly infection-related. RHD, which almost disappeared from the developed world, shows the most common pattern, which is related to untreated upper respiratory tract infection. Dilated cardiomyopathy in Malawi, which is mostly related to myocarditis given the young age of the patients and absence of any disease that could contribute to the left ventricular dilatation, is another example of “infection-related” CVD patterns. Furthermore, congenital heart disease could also be related to maternal infection during pregnancy. On the other hand, we did not find even a single case of documented CHD in the first 4 years of the study, i.e., from 2001 through 2004. The 3 documented CHD cases were identified in 2005. This could be interpreted as an emergence of degenerative-related CVD patterns in Malawi, which is another stage in the epidemiologic transition.

Interestingly, the CVD patterns in Malawi are to a large extent in agreement with the epidemiologic transition theory reported by Omran in the early 1970s.^6^ Considering that the most common patterns of CVD in Malawi reflect RHD, cardiomyopathies, and hypertensive heart disease, it is clear that Malawi is predominantly in the stage of receding pandemics. Nevertheless, the 3 recently documented cases of CHD could probably indicate the transition into the stage of degenerative and man-made diseases.

It seems that the epidemiologic transition in Malawi, and possibly in similar Sub-Saharan countries, is different from that which occurred in developed countries with regard to certain features. While in developed countries the spread of communicable diseases decreased and was replaced by a rise in the spread of non-communicable diseases, there was an overlap in Malawi, where there was a fairly significant prevalence of communicable as well as non-communicable diseases simultaneously. Moreover, the transition is occurring much faster in Malawi than it did in the developed countries. One reason for this may be the rapid economic growth and improvements in health care infrastructure that have occurred in parallel to the shift in the causes of ill-health and introduction of HAART for the treatment of HIV/AIDS. Strategies to control CVD in Malawi and similar developing countries must be based on these similarities and differences.

The limitations of this study are as follows: The distribution of CVD patterns in this work has been identified in a selective population (people referred to health care facilitates) in a country where there is inequity with regard to access to health facilities; this could be an important issue, which might have resulted in a selection bias. In this context, mild CVD patterns might have been reported rather than fatal CVD patterns. However, this might have been partially compensated by the fact that Mzuzu Central Hospital, where the survey was conducted, is the only major facility in the entire Northern Region. Therefore, it is likely that the CVD patterns reported in this study represent most of the actual CVD patterns. The interpretation of the results of this study in the context of epidemiological transition can only be speculated, considering the sparse data available on all the stages of epidemiologic transition in our study. Females constituted the majority of the study population. This may be due to differences in the perception of health among males and females or the increased proportion of females in the society.

## CONCLUSION

Malawi is in the stage of receding pandemics, which is characterized by CVD patterns predominated by RHD, cardiomyopathies, and hypertensive heart disease. Nevertheless, it is possible that Malawi is moving further into the stage of degenerative and man-made diseases. While the determinants of health transition in Malawi may be similar to those in developed countries, the dynamics are definitely different. The compressed time frame of health transition in Malawi imposes the large, double burden of communicable and non-communicable diseases, especially CVD. Hence organized efforts at documenting (the main purpose of this work), monitoring, and then preventing the emerging new patterns of CVD using resource-sensitive approaches is highly warranted.
